# Type A Cubital Venous Pattern among Students of a Dental College: A Descriptive Cross-sectional Study

**DOI:** 10.31729/jnma.6431

**Published:** 2021-06-30

**Authors:** Bipana Manandhar, Ritee Shrestha

**Affiliations:** 1 Department of Anatomy, Kantipur Dental College, Basundhara, Kathmandu, Nepal

**Keywords:** *cephalic vein*, *health professionals*, *prevalence*

## Abstract

**Introduction:**

Superficial veins in cubital fossa vary frequently in their anatomical pattern. The knowledge of variation of the cubital venous pattern is very essential for medical professionals for venous access during different medical procedures. This study aims to find the prevalence of Type A cubital venous pattern among students of a dental college.

**Methods:**

This descriptive cross-sectional study was conducted among 133 dental students of age 18 to 25 years in the department of anatomy of a dental college from November 2020 to February 2021. This study was conducted after obtaining ethical clearance from the Institutional Review Committee. Convenience sampling used and data was analyzed using Statistical Package for Social Sciences version 20. Point estimate at 95% Confidence Interval was calculated along with frequency and proportion for binary data.

**Results:**

Out of 133 dental students, Type A cubital venous pattern was found in 192 arms (72.18%) (64.56-79.79 at 95% Confidence Interval). Type A cubital venous pattern was seen in 116 (43.61%) left arms and 76 (28.57%) right arms of students. This pattern was observed in 139 (52.26%) females and 53 (19.92%) male arms respectively.

**Conclusions:**

This study showed higher Type A cubital venous pattern among dental students than other international studies.

## INTRODUCTION

The cubital fossa is a depression on the anterior aspect of the elbow. Main superficial veins in cubital fossa are cephalic, basilic and median veins. The cephalic vein takes its origin from the lateral aspect of the dorsal venous network of the hand and ends in the axillary vein. The basilic vein starts from the medial end of the dorsal venous network and forms an axillary vein. The medial cubital vein connects cephalic vein to basilic vein.^1^

These veins are commonly used for blood sampling, transfusions, and intravenous injections under emergency conditions. These veins are subjected to variation in their anatomic pattern. According to Del Sol, et al. cubital venous pattern is classified as Type A to Type F.^2^

This study aims to find the prevalence of Type A pattern of superficial veins in cubital fossa among dental students. Knowledge of the anatomic variation of these veins will be helpful for health professionals for easy access.

## METHODS

This was a descriptive cross-sectional study conducted on 133 students of 18-25 years from November 2020 to February 2021 in the Department of Anatomy, Kantipur Dental College Basundhara, Kathmandu. Ethical approval (reference number-36/020) was taken from Institutional Review Committee of Kantipur Dental College. Students with prominent veins and without any vascular diseases were included in the study.

Students with thick subcutaneous tissue and cut and wound in cubital region were excluded. Convenience sampling was done and sample size (n) was calculated as,

$$\begin{array}{ll} \text{n} & \text{= }{\text{Z}}^{\text{2}}\times \text{p}\times \text{q}/{\text{e}}^{\text{2}}\\ & \text{= }{\left(1.96\right)}^{\text{2}}\times 0.33\times (1-0.33)/{(\text{0.08)}}^{\text{2}}\\ & \text{= }132.715275\\ & =133 \end{array}$$

Where,

n = sample size
Z = 1.96 at 95% Confidence Interval
p = prevalence of Type A venous pattern from the previous study, 33%^3^
q = (1-p)
e = margin of error, 8%

Therefore the minimum required sample size was 133.

Type A cubital venous pattern resembles alphabet M. This pattern includes its subdivisions asType A1 in which median antebrachial vein divides into the median cephalic and median basilic veins, which join the cephalic and basilic vein respectively and Type A2 in which the median cephalic vein does not join with the cephalic vein. Venous patterns were recorded on both right and left arm along with name, sex and age of the student on a proforma. Type A venous pattern and its subdivisions Type A1 and Type A2 obtained were carefully studied and analyzed using Statistical Package for Social Sciences version 20. Point estimate at 95% Confidence Interval was also calculated.

## RESULTS

Out of 133 dental students Type A cubital venous pattern was found in 192 arms (72.18%) (64.56-79.79 at 95% CI). Among 133 students, 30 (22.55%) were male students and 103 (77.44%) were female students. The age range of the students was 18-25 years.

Among 103 (77.44%) females Type A pattern was seen in 139 (52.26%) arms, Type A1 pattern in 65 (24.44%) arms and Type A2 in 2 (0.75%). Among 30 (22.55%) males, Type A pattern was seen in 53 (19.92%) arms, Type A1 in 6 (2.25%) arms and Type A2 in 1 (0.37%) arm of students (Table 1).

**Table 1:** Distribution of observed cubital venous pattern in arms of male and female students (n = 266).

Cubital venous pattern	Male n (%)	Female n (%)	Total n (%)
Type A	53 (19.92)	139 (52.26)	192 (72.18)
Type A1	6 (2.25)	65 (24.44)	71 (26.69)
Type A2	1 (0.37)	2 (0.75)	3 (1.12)

Type A cubital venous pattern was 116 (43.61%) in the left arm and 76 (28.57%) in the right arm of students. Type A1 pattern was observed in 49 (18.42%) left arms and 22 (8.27%) right arms of students. The distribution of the Type A2 pattern was 2 (0.75%) and 1 (0.37%) in right and left arms respectively (Table 2).

**Table 2:** Distribution of observed cubital venous pattern in the right and left arm of students (n = 266).

Cubital venous pattern	Right arm n (%)	Left arm n (%)
Type A	76 (28.57)	116 (43.61)
Type A1	22 (8.27)	49 (18.42
Type A2	2 (0.75)	1 (0.37)

## DISCUSSION

Venous puncture is the commonly done procedure by healthcare professionals in cubital region where superficial veins are observed to be arranged in various pattern.^4^ The study of variation of cubital venous pattern is essential to assist medical and healthcare professionals to perform safer veni puncture. It helps to make aware of uncommon pattern of cubital veins which can avoid damage to neighbouring cutaneous nerves and arteries. Lack of knowledge can cause multiple punctures, subcutaneous hemorrhage and bruising.^5^

In our study, prevalence of Type A cubital venous patttern was 72.18% with 43.61% in left arm and 28.57% in right arm of students. Female students showed the higher incidence (52.26%) of Type A pattern than males (19.92%). The prevalence of Type A venous pattern of our study was slightly higher than the study conducted by Yammine Eric in which prevalence was 25%.^6^ Our study showed the higher incidence of Type A venous pattern in both males (19.92%) and females (52.26%) than the study carried out on the students of University of Jordan in which distribution of Type A pattern was 18.2% in males and 16.6% in females.^7^ The percentage of Type A pattern of our study in right (28.57%) arm was higher and left (43.61%) arm was lower than the percentage of the pattern in right (38.0%) and left (40.0%) arms of Chinese volunteers in a study carried out in Hospital Kuala Lumpur.^8^ Our study showed the higher incidence of Type A pattern than the study carried by Nicola et al in Department of Anatomy, Faculty of Medicine in Novi Sad, Serbia in which 34% arms of Caucasians with 53 right arms and 62 left arms showed the most common cubital pattern as Type A.^9^

In a study conducted on 170 males and 96 females selected from among the staff and students of the University Sains Malaysia's School of Medical Sciences in Kelantan, Malaysia revealed the distribution of Type A cubital patttern as 30.3%. Our study showed higher incidence of Type A pattern than their study. Our study also showed Type A pattern more frequent in females (52.26%) than in males (19.92%) which was in contrast with this study in which 18.8% Malays males and 11.5% Malays females showed this pattern.^10^ Our study was also of medical and paramedical stream of Rama Medical college, Mandhana, Kanpur in which incidence of Type A pattern was only 33.58%. The prevalence of Type A1 (26.69%) and Type A2 (1.12%) pattern of our study was slightly lower than the prevalence of Type A1 (29.54%) and Type A2 (4.04%) of this study.^11^ Our study showed slightly higher prevalence (72.18%) ofthis pattern than the study conducted on 536 Indian subjects which presented Type A pattern with incidence of 67.5%.^12^ This study was not similar with the study conducted in 800 upper limbs (200 men and 200 women) of a population in Bucaramanga, Colombiaas in which the distribution of Type A pattern was only 4%.^13^ This study was in contrast with the study conducted on 300 Chileans of Mapuche ethnic group of both sexes (30 men and 120 women) in which Type A pattern existed with the occurence of only 1%.^14^

The limitations of our study includes less number of malesin comparison to females. Larger sample with equal number of male and female students should be in further studies. Newer studies could be the cadaveric study for clear observation of the veins. Radiological techniques such as ultrasound could be used for clear visualization of these veins. It would be better if other parameters like length and diameter of veins could be measured.

## CONCLUSIONS

This study showed higher Type A cubital venous pattern among dental students. Knowledge of uncommon patterns of cubital venous patterns is very helpful for medical and paramedical health personnel to prevent injury to underlying structures and for correct intervention of these veins especially during emergency conditions.
